# Performance of the Wisconsin Card Sorting Test in Oncopediatric Patients in an Oncology Unit in Cali, Colombia: A Cross-Sectional Observational Study

**DOI:** 10.3390/children11070850

**Published:** 2024-07-13

**Authors:** Ángela María Jiménez Urrego, Valeria Santa, Manuel José Guerrero Gómez, Angie Carolina Guerrero Benitez, Tania Romo-González, Alejandro Botero Carvajal

**Affiliations:** 1 Faculty of Humanities and Social Sciences, Universidad de San Buenaventura de Cali, Cali 760036, Colombia; amjimenezu@usbcali.edu.co; 2 Department of Psychology, Universidad de San Buenaventura de Cali, Cali 760036, Colombia; vsantao@correo.usbcali.edu.co (V.S.); manuel.guerrero@u.icesi.com (M.J.G.G.); 3 Clinica de Occidente, Cali 760001, Colombia; angie.guerrero@clinicadeoccidente.com; 4 Institute of Biological Research, Universidad Veracruzana, Xalapa 91190, Mexico; tromogonzalez@uv.mx; 5 Faculty of Health, Universidad Santiago de Cali, Cali 760036, Colombia

**Keywords:** Wisconsin Card Sorting Test, oncology, neuropsychology assessment, executive function, pediatrics

## Abstract

Background: In 2020, the prevalence of cancer rose to 844,778 cases among the population aged 0–19 years. Approximately 90% of individuals under 18 years of age reside in low- and middle-income countries, where cancer survivors report adverse outcomes that negatively impact their general health, emotional state, and external factors such as academic performance due to the effect of these outcomes on executive functions. The Wisconsin Cart Sorting Test (WCST) is the gold standard for evaluating executive functioning. Therefore, this article (1) reports the performance of the Wisconsin Card Sorting Test (WCST) in oncopediatric patients from Cali, Colombia; (2) indicates the reliability of the WCST; (3) describes the association between cancer type and executive functioning in patients; (4) describes the differences between patients with various executive deficits and their executive total scores; and (5) describes the association between cancer type and the presence of brain deficits based on the WCST. Methods: In this cross-sectional observational study, 24 oncopediatric patients were interviewed and evaluated via the WCST. Results: The mean age was 12.08 years (SD 3.98); 20.8% of the patients were women, 70.8% had a primary diagnosis of leukemia, 8% exhibited acquired brain deficits, and more than 75% displayed adequate functional indicators of executive functions. Robust statistics were employed to explore the differences between the types of diagnosis and performance in executive functions, and no statistically significant differences were found (*p* = 0.156). We found that the WCST has a reliable Cronbach’s α of 0.804. Oncopediatric patients without brain deficits presented strong results in terms of executive functions (*p* = 0.002), with a moderate effect size (0.727). Conclusions: The WCST is reliable for discriminating executive functioning among pediatric cancer patients. The evidence suggests that there were no differences in the executive functioning of the participants based on the types of cancer being evaluated.

## 1. Introduction

Cancer is the second leading cause of death in the world. It accounted for a total of 10 million deaths, an incidence of 23.6 million cases, and 200 million disability-adjusted life years (DALYs) in 2019 [1]. North America has the highest cancer incidence among the population aged 0–19, with a total of 16,187 cases. Europe, Oceania, Latin America and the Caribbean, Africa, and Asia follow in that order, based on the size of the affected population. In 2020, the overall prevalence of cancer among patients in this age group was 844,778 cases, with leukemia being the most prevalent, accounting for 283,466 cases. This implies that approximately one child is diagnosed with cancer every minute [2,3]. In 2020, Colombia reported 2200 cancer cases among children and adolescents. The National Cancer Institute reported 204 new cases of childhood cancer between the ages of 0 and 18 years in 2022 and a survival rate that went from 40% to 61% [4,5,6]. The 5-year survival rate is greater than 80% in Europe and North America and close to 70% in Latin America [7]. However, cancer survivors have reported adverse consequences, including cognitive impairment that negatively impacts their health and academic performance [8].

The potential cognitive challenges that may result from oncological diagnosis and the numerous extant classifications are intriguing implications of cancer, particularly in pediatric oncology. The typical cognitive decline outcomes reported in cancer survivors include impairments in working memory, information processing speed, attention, and executive functions. This has a detrimental effect on academic performance in the immediate and long term through employment or educational support [8,9].

Ninety percent of the global population under 18 years of age resides in low- and middle-income countries, where they are subjected to adverse social factors such as poverty, inequality, and violence [10,11]. These factors contribute to the development of acquired brain problems that affect the brain functioning, quality of life, and mental health of children and adolescents [12]. The achievement of academic and work objectives depends, among other things, on the degree of behavior regulation and control. These functions are known as executive functions and have significant health implications [13,14].

The Wisconsin Card Sorting Test (WCST) is the gold-standard test used clinically because of its practical utility in identifying acquired cognitive problems linked to executive functioning performance [10]. Hence, clinicians frequently use the WCST to evaluate executive functioning in children with autism, offering a valid measure for determining the presence of reduced cognitive flexibility. In addition, it has been used for the evaluation of cognitive flexibility in children with attention deficit hyperactivity disorder. The relationship between this disorder and the tendency of infants to make perseverative errors has been shown to be a clinical aspect of low cognitive flexibility [15]. A previous validation study revealed that the WCST’s sensitivity increases from the age of 10 years onwards, due to the incipient maturational trait of cognitive flexibility in the brain of individuals aged 6–10 years [16]. It should be noted that the WCST has adequate internal consistency, inter- and intrarater test–retest reliability in the Colombian context, and validity regarding the construct of interest [15]. Furthermore, it has demonstrated a Cronbach’s alpha of 0.75 in the executive examination of Colombian individuals, indicating robust internal consistency and internal validity [16]. It has shown sensitivity regarding the executive functioning of neurotypical children and pathological diagnoses [17].

Pediatric patients with low-grade gliomas of the central nervous system undergoing surgical treatment, radiotherapy, or chemotherapy exhibit alterations in executive functions, memory, and motor skills; otherwise, these patients are children without oncological treatment or a diagnosis [18]. Thus, the evidence suggests that cancer diagnosis and/or treatment affects neurocognitive and executive functioning in survivors of childhood acute lymphoblastic leukemia [19]. In high-income countries, this concept has been extensively studied. However, in low- and middle-income countries, there is a scarcity of research in the field of mental health and developmental disorders [19,20]. This research contributes to five key findings: (1) it provides evidence on the number of oncopediatric patients with deficits in executive functioning; (2) it reports the reliability of the WCST in the oncopediatric population; (3) it indicates the association between the type of cancer and executive functioning; (4) it indicates the differences between patients with executive deficits and their total scores in executive functions; and (5) it shows the association between the type of diagnosis and the presence of acquired brain deficits based on the WCST results.

## 2. Materials and Methods

### 2.1. Context

The data were collected between 2021 and 2022. The city of Cali, Colombia has an approximate population of 13,942,630 minors as of 2024 [21], and it is the capital of the Department of Valle del Cauca, southeast of Bogotá. The rural population is admitted to the hospitals and clinics of Cali from peripheral departments and from the city of Cali for cancer care.

### 2.2. Participants

In addition to receiving palliative care, the study’s eligibility criteria included being a minor at the time of application, having an oncological diagnosis, and attending outpatient services for cancer treatment. We excluded those without the consent of their legal guardian. This study was conducted in accordance with the tenets of the Declaration of Helsinki for research on humans. Furthermore, the study also had the endorsement of the relevant Ethics and Research Committee.

This study included the entire database of pediatric patients. With a population size of 27 patients and an actual sample size of 24 patients, the study had its maximum error at a confidence level of 95% and a margin of error of 6.8%. Parental consent was obtained for all participants to participate in the study. In the outpatient clinic, the patient’s age was verified, the research team (A.M.J., M.G., and V.S.) was notified of the fulfillment of the inclusion criteria, and the study characteristics were explained to the participant and the legal guardian. Participants’ concerns were addressed, and informed consent was given with a signature.

The study included 27 patients. However, three protocols were discarded from the sample since these patients did not complete the test in its entirety, which impeded its scoring and rendered it impossible to determine the criteria used by the participants during each attempt.

### 2.3. Data Sources

Each patient’s information was obtained by interviewing the main caregiver and reviewing their medical history. Researcher A.M.J. and her team ensured that all data were anonymized and protected.

The following patient data were included in the study: identification label from 1 to 27 (all patients—including those excluded—were assigned a number for their anonymity); type of identification (civil registration or identity card); age, sex, department, city, educational link (studying or not), educational level (highest level reached), religion, health scheme (contributory or subsidized), diagnosis, type of diagnosis (large nosological groups), years since diagnosis, assessment, palliative care, radiotherapy treatment, caregiver role (role assumed; for example, mother, father, etc.); age of the caregiver, occupation of the caregiver, status of internment in the child protection system (in Colombia, this is the Colombian Institute of Family Welfare); type of family, family functionality, personal psychopathological history, treatment objective, patient location, laterality, correct responses (classification criteria correctly fed back); and complete categories, persistent errors and failures to maintain the attitude.

### 2.4. Procedure

The Wisconsin Card Sorting Test (WCST) was used due to its clinical utility, sensitivity, and validity in discriminating executive functions between clinical and nonclinical samples [13,14]. This test is used in research on pediatric psychiatric populations and is sensitive for detecting significant differences between pediatric patients with neurotypical and pathological neurologic disorders [19]. It exhibits validity and internal consistency for examining executive functions in Colombian individuals [15]. The test task involves classifying 128 cards based on the following classification criteria: color (the cards can be red, yellow, blue, or green), shape (triangle, circle, cross, or star), and number (of one to four figures per card). Since the task consists of choosing a criterion, waiting for feedback (correct or incorrect), and, in response to said feedback, either maintaining or altering the classification criteria, the WCST is the gold-standard test for evaluating the regulation and control of cognitive flexibility as domains of executive functions [18,22,23,24,25,26,27].

The literature suggests that executive functioning can be estimated with four indicators: correct answers, complete categories, perseverative errors, and failures to maintain one’s attitude. Each one independently provides information on an aspect of the executive functioning of the participant, which can be grouped around maintaining the action according to the objective of the task or, conversely, inhibiting actions unrelated to the objective.

The maintenance of the action is interpreted as follows: the higher the score, the greater the maintenance. The correct answer is the number of classification actions that were performed according to the stipulated classification criteria (shape, color, number), with a maximum score of 128. The number of classification criteria that have been fulfilled, with a maximum score of 6, is referred to as the “complete categories”. Conversely, maintenance failures are interpreted as follows: the lower the score, the greater the maintenance. Perseverative errors, which are repetitive actions unrelated to the current classification criteria, have a maximum score of 127. Similarly, the failure to maintain an attitude, which is an action that is consistent with the classification criterion that the participant fails to maintain, has a maximum score of 127 [10,20].

These scores assume that the subject may make an error after the criteria change or the initial feedback is given, according to the classification rule. Similarly, the typical score and classification can be estimated based on the child’s age, the educational level of the child, and the clinical or nonclinical group (Table 1).

Classification is based on the direct score obtained in the WCST. Note: In this research, scores between 0 and 84, according to the manual, were assumed to indicate brain deficits. Scores between 85 and 107+ were considered to indicate expected age-appropriate brain function.

The application and qualification of the instrument were carried out by A.B.C., V.S., and M.G., who are trained in neuropsychology. The researchers randomly evaluated the protocols twice in accordance with the WCST’s application and qualification guidelines.

The data were processed in the Jamovi software version 2.3. Qualitative variables were expressed as frequencies and percentages, and quantitative variables were expressed as means and standard deviations. Hypothesis tests were performed to verify normality assumptions for the quantitative variables. Since the Shapiro–Wilk test indicates deviations from the normality of the data together with the small sample size, robust statistics were used in the Walrus module, and the trimmed mean was indicated.

The Mann-Whitney U test was employed to compare total executive functioning between patients with and without brain deficits according to the WCST. The hypothesis was accepted with a value of *p* < 0.05 and an effect size of 95% CI. The total executive function was estimated according to Kopp [14] as the sum of the four indicators (correct answers, complete categories, perseverative errors, and failures to maintain an attitude). Since the last two indicators are inversely related to executive function, that is, a lower score translates to greater performance, perseverative errors multiplied by minus one were added, and failures for the maintenance of attitude were multiplied by minus one. The reliability of the instrument for this sample was estimated using Cronbach’s alpha.

To evaluate the differences between groups according to the type of diagnosis, the Kruskal-Wallis test [28,29,30,31,32,33] was used.

## 3. Results

### 3.1. Characteristics of the Participants and Executive Functioning

A palliative care protocol was implemented for twenty-four oncopediatric patients, who continued to follow their treatment regimen. Table 2 illustrates the baseline data of the participants.

WCST performance revealed 8 patients with executive deficits in the sample of 24 participants. The mean total executive function in the group of 24 patients was 54.25 (SD 27.61), which was considered a relatively low score for the group of oncopediatric patients. However, no deficits were observed in the four executive function indicators in more than 75% of the participants.

The instrument’s reliability in the sample was satisfactory, as evidenced by a Cronbach’s α of 0.804.

### 3.2. Association between Type of Cancer Diagnosis and Executive Function Performance

Table 3 shows the associations between the types of diagnoses and the total scores on each of the WCST subscales.

There was no association between the type of oncological diagnosis and the total performance of executive functions (*p* = 0.156). The two dimensions that make up the scale of executive functions—inhibition of action and maintenance of action—were consistent with the results obtained for the total scale. These values were not significant (*p* = 0.139 and *p* = 0.203, respectively). We found a statistically significant value (*p* = 0.044) in complete categories, which implies that there is an association between the type of diagnosis and the maintenance of an action; however, the effect size was small (ε^2^ = 0.351). The two-by-two comparisons of Dwass–Steel–Critchlow–Fligner indicate that there were no differences in the comparisons between couples, as is the case with the associations among lymphomas–leukemias, leukemias–bone tumors, and leukemias–solid tumors.

### 3.3. Differences in Executive Function between Patients with Executive Deficits and Total Scores

We explored the differences between the patients who, according to the WCST score, presented with executive deficits and the total score on the EF scale. Given that there is ample evidence about low performance in executive functions when executive functioning deficits are present, the hypothesis that the group without deficits in these functions would have a higher total score in executive functions was tested (Table 4).

**Figure 1:**
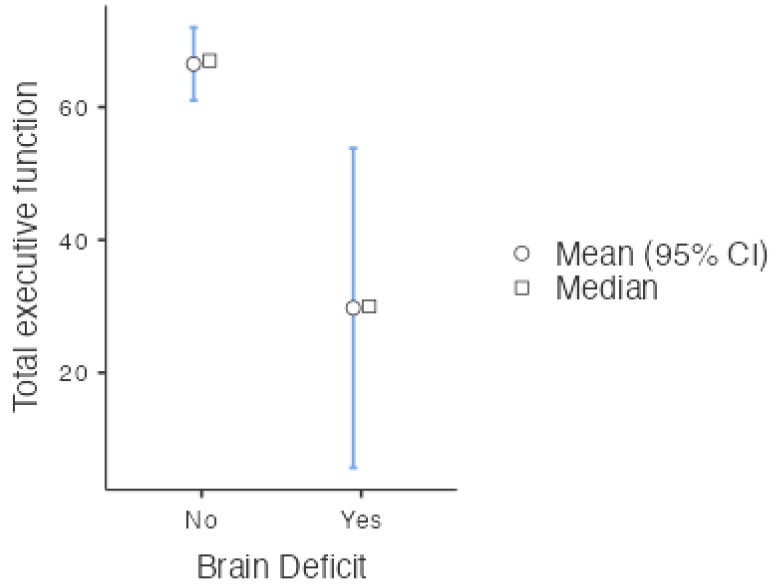
Descriptive plot: differences in medians between the nonsurgical groups.

There was a statistically significant difference between the two groups of patients (*p* = 0.002), and the effect size was moderate (0.727). This implied that the patients who presented with brain deficits according to the WCST had lower executive function scores, and this difference was not due to chance (Figure 1).

### 3.4. Association between the Type of Oncological Diagnosis and Brain Deficit According to the WCST

Lastly, we explored the association between the presence of acquired brain deficits and the type of diagnosis among cancer patients, as determined by the WCST (Table 5).

Fisher’s exact test revealed no association (*p* = 0.690) between the type of diagnosis and the acquired brain deficit, and the strength of the association in the sample was weak according to the contingency coefficient of 0.285.

## 4. Discussion

We present the executive function performance of 24 pediatric cancer patients from a middle-income country. This report is organized into five key findings: (1) The WCST is a reliable instrument for measuring executive functions in the oncopediatric population; (2) performance in executive functions is not associated with the type of cancer; (3) eight patients presented with alterations in executive functioning; (4) there are differences between patients based on their executive deficits and their total scores; and (5) there is an absence of associations between the type of diagnosis and the presence of brain deficits according to the WCST.

Due to the fact that the brain continues to develop until the age of 20 and is more susceptible to environmental and hereditary agents that influence the maturation process, we considered identifying a greater number of patients with unfavorable executive functioning. Consequently, 70% of oncopediatric patients experience white matter destruction as a result of toxic side-effects during cancer treatment [34,35,36,37,38]. This adverse effect on cognitive functioning has been evidenced by increased challenges in written communication, visual memory, cognitive flexibility, and the ability to plan [39,40]. In addition to moderate and severe difficulties in attention, processing speed and perceptual reasoning directly affect the learning process of children during their school years [41]. The degree of involvement depends on age, family history, the type of cancer present, and the dose administered to treat the cancer [39,40,41].

Although the performance of the WCST in oncopediatric patients allowed for a cross-sectional observation of 24 children in this study, the results obtained showed relatively low executive functioning in general, which is consistent with research in high-income countries [42].

This behavior is observed among cancer survivors due to the vulnerability of executive functions and processing speed to treatment, which alters brain function and structure [42,43]. These cognitive deficits are related to alterations in the gene expression of microglia in the prefrontal cortex. This alteration in gene expression has been associated with a cancer diagnosis at an early age and exposure to chemotherapy [44]. Although the present study did not find a relationship between the type of cancer diagnosis and the overall performance of executive functioning (*p* = 0.156), a significant relationship was found between the type of diagnosis and the number of categories completed (*p* = 0.044; ε^2^ = 0.351).

The completed categories allude to the ability of the participants to maintain an action. This is important because it is a useful predictor of the executive memory performance of each individual. Although the WCST instrument was designed to assess executive functioning, it has also been demonstrated to be useful in relating executive functions to other aspects of human cognition. For example, it has been contrasted with the Wechsler III Memory Scale. This emphasizes its potential as a predictor of advanced reasoning skills, concept formation, and set-shifting, as well as for measuring information processing and storage [45]. The test is highly sensitive in the measurement of executive and cognitive dysfunction [46], in addition to its usefulness in cognitive evaluations of patients with schizophrenic disorders and psychoactive substance abuse [47].

Despite the size of the effect of the aforementioned relationship (ε^2^ = 0.351), it was observed that oncological diagnosis is linked to working memory and executive function performance in cancer patients unrelated to the central nervous system involving neuroinflammatory processes and oxidative stress [48].

This phenomenon is equally attributable to the secondary cognitive impairment of chemotherapy and radiotherapy in cognitive domains [46], including working memory and poor performance in frontal lobe tests [49]. Conversely, McDonald and colleagues discovered hyperactivity in frontal areas using fMRI when evaluating the working memory of patients with breast cancer in contrast to a control group after receiving chemotherapy for more than two years. This finding clarifies the role of diagnosis that is not related to the central (peripheral) nervous system, as well as its treatment, by indicating the presence of compensatory hyperarousal in brain circuits associated with working memory [50].

The type of cancer has been shown to be an indicator of cognitive decline [51,52]. Wouters and colleagues have reported that lymphoma diagnosis in adults tends to be associated with cognitive decline in 16% of clinical samples (*n* = 106), with at least 1.5 standard deviations below the control group (*n* = 56). This is despite the absence of significant neuropsychological differences between their clinical group and control group [53].

In this context, it is pertinent to acknowledge the existence of cognitive deficits as a phenomenon that exhibits a certain degree of association with peripheral tumors and their treatment. Moreover, working memory dysfunction has been observed in children with acute lymphoblastic leukemia compared with children without acute lymphoblastic leukemia, especially in terms of correct answers and precision. Defects were also observed in executive functioning and cognitive flexibility, as measured by the WCST [54].

However, the present study, as well as the antecedents presented, underscores the importance of examining the scarce association between peripheral diagnostic typologies as a predictor of brain deterioration or dysfunction. This indicates the relevance of longitudinally assessing the effects of cancer treatment according to its variant to contrast its role with the pediatric population.

Despite this premise, the ability of the WCST to expand the available knowledge about its construct validity was tested. We verified that there were statistically significant differences in executive functioning between the patients with and without brain deficits (*p* = 0.002), with a moderate effect size (0.727).

This phenomenon highlights the existence of a deficit in executive performance in the current sample and enables us to evaluate the role of familial, social, or educational variables that contribute to such cognitive deficiencies. This is based on variables in addition to cancer treatment, which has been shown to influence cognitive deterioration according to its typology given the neurotoxic effects of chemotherapy, such as methotrexate [55]. In addition to demonstrating that survivors of pediatric cancer are at an increased risk of experiencing mental health problems and cognitive dysfunction, variables such as lifestyle, lack of care for medical factors, experimentation with distress, genetics, and molecular risks have been identified as risk factors for cognitive impairment associated with cancer [56].

Krull and colleagues have longitudinally reported cognitive dysfunction in most cognitive and behavioral domains in patients surviving acute lymphoblastic leukemia 26 years after diagnosis. These authors noted that deficits tended to occur in participants exposed to chemotherapy and low doses of cranial radiation therapy. They also associated neurocognitive impairment with variables such as full-time employability and schooling. It was revealed that treatment initiated at an earlier age resulted in more progressive impairments in cognition, memory, and academic performance over time. [57]. Thus, it is crucial to consider the influence of these variables on brain dysfunction in pediatric and adult oncology for the purposes of the present study.

It is pertinent to note the reliability of the WCST in evaluating executive dysfunction in patients in the present study. This finding coincides with prior research that has investigated the reliability and sensitivity of this test to determine the presence or absence of deficiencies at the executive level [10,30,31]. This information prompts us to discuss the criticisms that the instrument has received regarding the limitations of its ecological validity [46,49,50] and the importance of improving the latter in the executive evaluation process [51]. Regarding the psychometric properties of the WCST, a recent validation study in the Colombian context characterized the WCST’s adequate internal consistency and inter- and intrarater test–retest reliability in comparison to those of classic batteries such as the Neuropsi and BANFE 2. These authors reported validity regarding the construct of interest for the WCST. However, the findings of these authors were known for their failure to demonstrate a correlation between this test and the reference measurements of frontal functions made with the BANFE 2 [16].

It is pertinent to mention that the present research was carried out in an environment where the environmental control of variables was reduced. This was a result of the obstacles involved in the field work typical of the hospital setting, where various alterations were found in the patients’ life dynamics, including those related to their family, social, school, and disease-specific dimensions, which can influence the oncopediatric process. However, our group will elaborate on this interaction between the oncopediatric aspects and the narratives of caregivers about the family process in future articles.

Despite the progress made in the treatment of this disease, cancer patients continue to experience a variety of secondary effects in the short, medium, and long term. These effects are primarily the result of the various processes and transformations that are employed to combat the disease. Short-term physical effects, such as nausea, pain, fatigue, weight loss, and hair loss, develop during treatment; however, they subside upon the completion of all procedures. Conversely, medium-term effects are those that appear during or after treatment, such as impairments in cognitive functions and/or processes. Finally, late side-effects are symptoms that manifest months or years after treatment, including heart failure or second cancers. Therefore, it is crucial to continue to investigate and monitor the neuropsychological and psychological processes of patients who undergo this diagnosis in order to safeguard their quality of life.

We are careful about generalizing our findings to neuropsychology and psychology [58]. We believe that this study serves as a foundation for describing the WCST’s reliability as a diagnostic and complementary tool in palliative care services for the oncopediatric population in middle-income countries like ours. This is due to the impacts of treatment and diagnosis on brain function and executive functions, as well as their future outcomes in terms of academic and/or career performance.

## 5. Conclusions

The WCST is reliable for evaluating executive functioning in oncopediatric patients. Differences in executive performance were not associated with the type of oncological diagnosis or with acquired brain deficits, according to the WCST.

The use of the WCST in this sample enabled the identification of low-socioeconomic-status patients with an acquired brain deficit that can modulate brain function, executive functions, and health outcomes.

A case–control study is suggested for future work in order to evaluate differences in executive functioning between children exposed to cancer and children without such exposure.

## Figures and Tables

**Table 1:** Interpretation according to performance in the WCST.

Classification	Threshold	Score
Significantly deficient	Severe impairment	0–54
	Severe to moderate impairment	55–61
Poor	Moderate deterioration	62–69
Borderline	Moderate to intermediate	70–76
	Intermediate deterioration	77–84
Expected	Below average	85–91
	Medium	92–106
	Above average	107+

**Table 2:** Sociodemographic characteristics and performance of the study participants in the WCST.

Overall	
*n*	24
**Age (mean (SD))**	12.08 (3.98)
**Sex: Female *n* (%)**	5 (20.8)
**Department (%)**	
Cauca	10 (41.7)
Caqueta	2 (8.3)
Cauca Valley	12 (50.0)
Study: *n* (%)	5 (20.8)
Health scheme ^1^: Contributory *n* (%)	5 (20.8)
**Diagnostic Typology (%)**	
Leukemias	17 (70.8)
Lymphomas	3 (12.5)
Bone tumors	3 (12.5)
Solid tumors	1 (4.2)
**Cognitive Profile of the Sample—WCST Results**	
Laterality: Left-handed (%)	2 (8.3)
Correct answers (mean (SD))	74.12 (13.02)
Response deficit: *n* (%)	18 (75.0)
Complete categories (mean (SD))	4.46 (1.79)
Deficit categories: *n* (%)	18 (75.0)
Perseverative errors (mean (SD))	22.75 (16.17)
Perseverative error deficit: *n* (%)	18 (75.0)
Maintain attitude (mean (SD))	1.58 (1.72)
Maintaining attitude deficit: *n* (%)	20 (83.3)
Maintenance action (mean (SD))	78.58 (14.19)
Action inhibition (mean (SD))	−24.33 (15.95)
Total executive function (mean (SD))	54.25 (27.61)
Executive functioning deficit: Yes (%)	8 (33.3)

^1^ The health regime in Colombia is publicly (subsidized) and privately (contributory) funded.

**Table 3:** ANOVA of Kruskal-Wallis factors.

Subscale and Total	χ^2^	gl	*p*	ε^2^
Total executive function	5.23	3	0.156	0.227
Inhibition action	5.50	3	0.139	0.239
Maintenance action	4.61	3	0.203	0.200
Maintain attitude	3.02	3	0.388	0.131
Perseverative mistakes	5.04	3	0.169	0.219
Complete categories	8.08	3	0.044	0.351
Correct answers	4.07	3	0.254	0.177

**Table 4:** Independent sample *t*-test.

		Statistic	*p*		Effect Size
Total executive function	Mann-Whitney U	17.5	0.002	Rank biserial correlation	0.727

**Table 5:** Contingency table by type of diagnosis and brain deficit.

Type of Diagnosis		Brain Deficit		Total
		No	Yes	
Leukemias	Observed	12	5	17
	% within row	70.6%	29.4%	100.0%
Lymphomas	Observed	1	2	3
	% within row	33.3%	66.7%	100.0%
Bone tumors	Observed	2	1	3
	% within row	66.7%	33.3%	100.0%
Solid tumors	Observed	1	0	1
	% within row	100.0%	0.0%	100.0%
Total	Observed	16	8	24
	% within row	66.7%	33.3%	100.0%

## Data Availability

This manuscript is under the review of Mendeley Data, and the data are available in brief.
